# Two-stage revision anterior cruciate ligament reconstruction

**DOI:** 10.1186/s43019-019-0010-6

**Published:** 2019-09-18

**Authors:** Du-Han Kim, Ki-Cheor Bae, Dong-Wan Kim, Byung-Chan Choi

**Affiliations:** Department of Orthopaedic Surgery, Dongsan Medical Center, School of Medicine, Keimyung University, 1035 Dalgubul-ro, Dalseo-gu, Daegu, 42601 South Korea

**Keywords:** Knee, Anterior cruciate ligament, Reconstruction, Revision, Stage

## Abstract

With the rising number of anterior cruciate ligament (ACL) reconstructions, revision ACL reconstructions are becoming increasingly common. A revision procedure may be performed to improved knee function, correct instability, and facilitate a return to normal activities. When performing a revision reconstruction, the surgeon decides between a single-stage or a two-stage revision. Two-stage revisions are rarely performed, but are particularly useful when addressing substantial tunnel-widening, active infection, and concomitant knee pathology (e.g., malalignment, other ligamentous injuries, meniscal or chondral lesions). Among these potential scenarios requiring a two-stage revision, tunnel-widening is the most common cause; the first stage involves graft removal, tunnel curettage, and bone grafting, followed by revision ACL reconstruction in the second stage. The purpose of this article is to review the preoperative planning, surgical considerations, rehabilitation, and outcomes of two-stage revision ACL reconstructions and summarize the recent literature outlining treatment results.

## Background

Anterior cruciate ligament (ACL) reconstruction rates have increased over the past 20 years to roughly 200,000 per year [1]. As this number has continued to increase, the incidence of revision ACL reconstruction (ACLR) has also grown to a rate of between 4.1 and 13.3% of all primary ACLRs performed [2]. The goal of revision ACLR is to improve knee stability and activity levels, but the outcomes are reported to be inferior to those of primary ACLR [3]. Successful revision surgery requires an understanding of the cause of failure, careful preoperative planning, meticulous surgical execution, proper postoperative rehabilitation, and appropriate patient counseling [4].

Revision ACLR surgeries can be mainly divided into one-stage and two-stage procedures. Two-stage revision ACLR typically involves an initial bone-graft procedure—to fill the widened or misplaced tunnels—and subsequent time to allow for the bone graft to heal sufficiently before the second stage is undertaken [5]. A relatively small but challenging subset of patients requires two-stage revision ACLR. Reports suggest that a two-stage procedure is performed in only 8 to 9% of revision ACLRs [6].

To date, the literature on revision ACLR surgery has primarily focused on comparing the outcomes to those of primary ACLR. While one-stage revision ACLR is well described and reported, few studies have reported the outcomes of two-stage revision ACLR. For the aforementioned reasons, in this review, we will provide an overview of two-stage revision ACLR in the following order: preoperative planning, surgical considerations, rehabilitation, outcomes, and conclusions.

## Preoperative planning for two-stage ACLR

Preoperative planning for revision ACL surgery is essential for a successful outcome. The important stages in assessing a patient with failed ACL surgery include history, patient selection, physical examination and investigations, choice of graft, surgical technique, and rehabilitation [7]. Major reasons to proceed with a two-stage strategy include tunnel-widening or other loss of bone stock, tunnel malposition, arthrofibrosis, active infection, concomitant meniscal deficiency, malalignment, and focal chondral lesions and/or other ligamentous laxity that may require a staged approach [8, 9] (Table 1).

**Table 1:** Indications for two-stage revision anterior cruciate ligament reconstruction

Indications	
Tunnel-widening (10–15 mm)	
Tunnel malposition that precludes avoidance of primary tunnels	
Active infection	
Arthrofibrosis (loss of more than 5° of extension or 20° of flexion)	
Meniscal deficiency and/or chondral lesion	
Malalignment (including an increase in posterior tibial slope)	
Other ligamentous laxity	

An active infection should be treated with irrigation and debridement with confirmation of eradication (e.g., normalized laboratory test results, negative cultures) before a patient has a new graft and implant put in place. Similarly, a patient with a loss of more than 5° of extension or 20° of flexion of knee motion should be considered for lysis of adhesions and manipulation under anesthesia followed by rehabilitation [4, 10].

Tunnel orientation and size are the most important causes related to the two-stage procedure, as these enlarged tunnels may complicate graft placement and fixation [11, 12]. Although there are many proposed theories for tunnel lysis, it is most accurate to state that this condition has a multifactorial origin; mechanical and biologic causes have been reported, and both contribute to enlarged graft tunnels [11, 13]. Tunnel malpositioning that will interfere with new revision reconstruction tunnel placement can reduce graft apposition within the tunnels at the time of graft fixation, thereby placing the graft stability and subsequent incorporation at greater risk of failure [11].

**Fig. 1:**
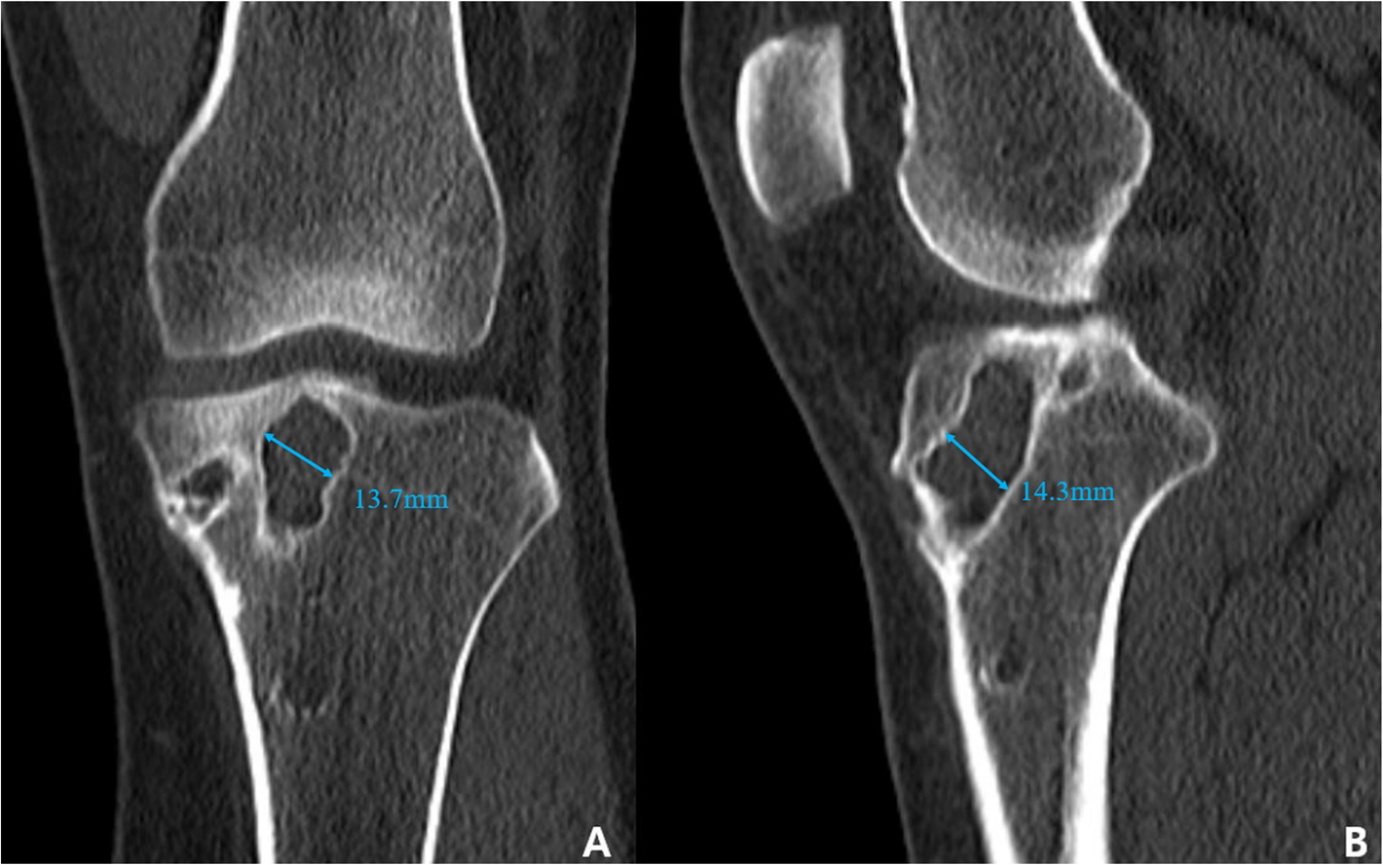
Coronal (**a**) and sagittal (**b**) view of computed tomography (CT) images demonstrate widening of the tibial tunnel in the setting of a failed anterior cruciate ligament reconstruction. Measurements are made perpendicular to the axial plane of the tunnel at the widest point

Currently, the “gold standard” for measuring tunnel size is the computed tomography (CT) method. Studies have shown that CT outperforms magnetic resonance imaging (MRI) and radiographs in both inter- and intra-observer reliability for evaluating tunnel-widening [14, 15]. When measuring with CT, the axial-plane image is considered incorrect because the plane of cuts is inconsistent. Therefore, the coronal and sagittal images (four-tunnel view; femur-coronal, tibia-coronal, femur-sagittal, tibia-sagittal) are primarily used (Fig. 1). Measurements are made perpendicular to the axial plane of the tunnel at the widest point [15].

Previous literature has reported that if the tunnel size exceeds 10–15 mm, two-stage surgery should be performed. However, an absolute threshold for how much tunnel-widening and bone loss is acceptable to undergo a single stage with an intraoperative bone graft prior to drilling has not been established [4, 16–19]. Battaglia and Miller [12] indicated that bone grafting should be performed in cases with a tunnel diameter of 10–15 mm. Additionally, Brown and Carson [20] regarded patients with a bone tunnel of < 15 mm diameter as good candidates for grafting. They explained that because a bone tunnel of 15 mm diameter with 45° of inclination resulted in a tibial tunnel aperture of > 20 mm, a 20-mm tunnel aperture was regarded as a candidate for grafting. Yoon et al. [21] evaluated 88 patients who underwent one-stage revision ACLR. The patients were divided into two groups based on the tunnel diameter (group A, < 12 mm; group B, < 12 mm). At a mean follow-up of 7.9 years, clinical scores following revision ACLR did not differ significantly according to the tunnel size. However, the results of the postoperative side-to-side differences of the Lachman test as well as the pivot-shift test were significantly superior in group A (< 12 mm).

## Surgical considerations of two-stage ACL reconstruction

### Bone grafting

Autograft bone, either from the iliac crest or anterior tibial plateau, is still considered the gold standard source for grafting because of its osteoconductive, osteoinductive, and osteogenic properties. Clinically, many authors have reported good results for two-staged revision ACLR using autograft bone [4, 11]. Thomas et al. reported that the laxity measurements achieved with a two-stage revision ACLR using autograft iliac bone could be similar to those achieved after primary ACLR and clinical improvement [11]. But an iliac-crest autograft is comparatively invasive with relatively high donor-site morbidity and the potential for insufficient yield quantities [11, 22]. For an allograft, a single bone dowel approximately 1 mm larger than the diameter of the tunnel is used and placed using a bone tamp for a press-fit technique, ensuring that the entire tunnel is filled [4]. The use of allograft material negates the issue of donor-site morbidity but carries the potential risk of disease or infection transmission [23, 24]. To minimize the risk of viral and bacterial contamination, allograft bone is sterilized. However, methods used to sterilize allograft material (e.g., gamma irradiation and autoclaving), are known to adversely affect the structural and other properties of the graft material [25].

Recently, a technique for sterilizing musculoskeletal allografts using supercritical carbon dioxide (sCO2) has been developed [26]. In theory, the sCO2-sterilized graft only provides osteoconductive properties to the grafted bone tunnels. The metaphyseal location and predominantly cancellous bone surrounding the graft tissue result in high osteoinductive and osteogenic potential from the host’s bone marrow [26]. Van de pol et al. [26] reported the use of a sCO2-sterilized bone allograft to fill tunnel defects as the first stage of a two-stage revision ACLR. The mean time between the two stages was 8.8 months and in the second stage, bone-biopsy specimens were taken from the tibia. They found that a sCO2-sterilized bone allograft showed graft incorporation and remodeling through creeping substitution.

Silicate-substituted calcium phosphate (Si-CaP), which represents a synthetic, porous bone-graft substitute, may also be an appropriate bone-graft substitute [27–30]. Si-CaP appears to provide a more stable osteoconductive scaffold to support faster angiogenesis. Von recum et al. [31] used Si-CaP for a bone-graft substitute for tunnel augmentation in two-stage revision ACLR. Punch-biopsy specimens of the augmented tunnels were taken at the two-stage procedure, and histologic examination included quantitative analysis of the area of immature bone formation, lamellar bone, and bone marrow. CT analysis also included the determination of the filling rates of the tunnels. They reported that Si-CaP as a bone-graft substitute for tunnel augmentation showed favorable histologic, radiologic, and intraoperative integration comparable to the autologous iliac bone graft.

### Timing of two-stage revisional ACL reconstruction

The optimal and earliest possible timing of the two-stage procedure is still not clear. Typically, a staged procedure requires an average delay of 4 to 6 months to allow for the bone defect to heal [11, 18], likely subjecting patients to a prolonged period of knee instability and thus adding to the risk of meniscal injury, additional deterioration of muscle strength, and osteochondrosis [32]. For assessment of bone-graft incorporation, radiographs are routinely used. Some authors have described the additional use of CT scans to confirm healing at 3–5 months after bone grafting [4, 12, 33, 34]. Thomas et al. performed a CT scan at 4 months to assess healing of the bone graft in the tibial tunnel. Blurring of the tunnel margins, reactive sclerosis, and the presence of bone within the tunnel were used as signs of adequate healing. They observed that an average of 5.8 months was needed for healing of the autograft dowel to become visible on CT scans [11]. Uchida et al. [34] reported 10 consecutive patients (four female and six male patients with a mean age of 28 years) who underwent autogenous bone grafting prior to ACLR revision. CT examinations were performed at 3, 12, and 24 weeks after bone grafting. Evaluations were performed in the axial plane of the tibia using three parameters (occupying ratio, union ratio, and bone mineral density). They recommended that two-stage reconstruction could be safely performed at 24 weeks after bone grafting by the iliac-bone block-grafting technique.

### Graft choice and fixation

There has been a long-standing debate as to whether an autograft or an allograft should be used for revision ACLR. A decision that will often depend on the graft used during the primary ACLR. However, many authors prefer using an autograft for revision ACLR when possible. According to the result of the multicenter ACL Revision Study (MARS) Group, the risk of graft re-rupture following revision ACLR in patients receiving an autograft is 2.78 times less likely than in those receiving an allograft [35]. Noyes et al. advocate that the allograft should not be considered as the first choice of graft for revision surgery [36]. If no autograft is available for revision surgery, they advise augmentation of the allograft with the lateral extra-articular iliotibial band procedure to reduce the high failure rate associated with the use of the allograft.

Patient age and activity level are also important factors when deciding on graft choice for revision procedures. Allografts may be well suited for recreational athletes older than 30 years of age, but autografts may be a better choice for younger athletes who wish to return to higher-level athletics [4].

Secure graft fixation is critical in ensuring a successful two-staged ACLR. Because of weak bone from bone-grafted tunnels or enlarged tunnels, the surgeons should pay careful attention to the fixation methods and consider double fixation in all revisions [37]. The insertion of an interference screw not only compresses the graft in the tunnel but also leads to an enlargement of the bone tunnel itself [13]. When aperture fixation is not possible, familiarity with, and use of, all-inside tibial and femoral sockets with cortical suspensory fixation may be necessary [4].

### Additional procedure

Numerous studies have reported that additional procedures (e.g., extra-articular tenodesis, anatomical anterolateral ligament (ALL) reconstruction) could be a meaningful option in cases of revision ACLR to improved rotatory stability which is associated with re-injury.

Trojani et al. [38] have reported the outcomes of revision ACLR with and without lateral extra-articular tenodesis. They noted that although additional lateral tenodesis did not influence the International Knee Documentation Committee (IKDC) score in a multicenter study of 163 revision ACLRs, the proportion of negative pivot shifts was 80% with lateral tenodesis plus revision ACLR versus 63% without tenodesis. Louis et al. [39] have demonstrated that 349 patients who underwent revision ACLR-combined-ALL reconstructions showed improving rotational stability without increasing the risk of early and late complications and the re-rupture rate was 1.2% in their multicenter study.

Lee et al. [40] reported the results of 87 patients who underwent revision ACLR with a follow-up of more than 3 years. Patients were divided into the isolated revision ACLR group (*n* = 45) and the revision ACLR group in combination with ALL reconstruction (*n* = 42). They observed that revision ACLR in combination with ALL reconstruction significantly reduced rotational laxity and showed a higher rate of return to the same level of sports activity than revision ACLR alone, although there were no significant differences in anterior laxity or functional test results between the two groups.

## Rehabilitation

In the immediate postoperative period, the weakest part of any ACLR is the fixation. After 6 to 12 weeks, failures tend to occur in mid-substance [11]. Some authors suggest that an accelerated rehabilitation program for revision ACLR is not appropriate because of weaker initial graft fixation [20]. However, Thomas et al. [11] noted that this suggestion is unnecessary, as using a two-stage technique ensures that there is good-quality bone around the tunnels, and the initial graft fixation is as secure as in the primary reconstruction.

Rehabilitation after the initial bone-grafting stage shares similarities with standard ACLR protocols [17]. The initial rehabilitation emphasis is focused on restoring tibiofemoral and patellofemoral passive range of motion, restoring quadriceps’ activation, and controlling and resolving any joint effusion. No restrictions are placed on their range of motion and patients were allowed to weightbear on the affected leg using crutches [17]. Physical therapy with muscle-strengthening and proprioceptive training can be performed. Improved muscle strength may be the decisive factor; however, changes in functional movement patterns after intensive physical therapy are also important to consider [41].

## Outcomes

Few studies report the outcomes of two-stage revision ACLR alone. Current studies report an average-low failure rate of 3.6% (wide range of 0–8.1%) for utilizing two-stage revision ACLR [11, 33, 34, 42, 43] (Table 2).

**Table 2:** Current reports on outcome of two-stage revision anterior cruciate ligament reconstruction

Year	Author	Level of study	Country	No. of case	Mean age (years)	Bone graft	Mean F/U	Outcomes
2005	Thomas et al.	III	United Kingdom	49	35.4	Iliac autobone	6.2 years	IKDC: 61.2, Objective laxity: 1.36 mm
2013	Franceschi et al.	IV	Italy	30	29.1	Proximal tibia autobone	6.7 years	Lysholm: 90.2, return to sports: 66.7%
2016	Uchida et al.	IV	Japan	10	28.0	Iliac autobone	Minimum 2 years	Negative instability test, Lysholm: 96.6
2017	Mitchell et al.	III	USA	49	30.4	The Opteform allograft bone	3.1 years	Failure rate: 6.1%, Lysholm: 77
2018	Diermeier et al.	III	Germany	44	30.5	Iliac autobone	33.9 months	Lysholm: 77.2, IKDC: 69.0, Tegner: 4.1

Values are presented as number only, mean standard deviation, or mean only

*F/U* Follow-up, *NA* Not available, *IKDC* International Knee Documentation Committee

Thomas et al. [11] reported the results of 49 consecutive two-stage revision ACLRs in which the tibial tunnel was grafted (the bone graft was taken from the ipsilateral iliac crest) during the first stage, followed by an ACLR using various grafts and fixation methods for the second stage. The results from this group were compared to the results of a matched group of patients with primary ACLR. The two-stage group contained significantly more patients with meniscal and chondral pathology compared with the primary ACLR group. At a mean follow-up of 6 years, the laxity measurements achieved with a two-stage revision ACLR can be similar to those achieved after primary ACLR, although the IKDC rating is lower.

Franceschi et al. [33] evaluated 30 patients who underwent two-staged ACLR revision procedure after a traumatic re-rupture of the ACL. All the patients in the study underwent screw removal and filling of the tunnels with an autograft harvested from the anterior tibial metaphysis. The second stage of the revision ACLR was performed a minimum of 3 months later, after obtaining a CT demonstrating adequate filling of the tunnels using a hamstring autograft though a transtibial drilling technique. The new ligament was fixed to the tibia by a metallic screw and to the femur by a bioabsorbable screw. At a mean follow-up 6.7 years postoperatively, 66.7% of patients had returned to their preoperative sports activity level, 23.3% had changed to lower, non-impact sports, and 10% had given up any sports activity. There was also a significant improvement in the Lysholm score when comparing preoperative and postoperative values.

Uchida et al. [34] evaluated 10 consecutive patients who underwent staged revision ACLR using autogenous bone grafting and reported that all patients had a full range of motion of the knees, a negative Lachmann sign and negative pivot-shift test . An average Lysholm score at 2 years post operation was 96.6 points ± 2.1 (91–100 points).

One comparative cohort study reported that objective outcomes and subjective patient scores and satisfaction were not significantly different between one-stage and two-stage revision ACLRs and both groups had significantly improved objective outcomes and patient subjective outcomes without notable differences in failure rates [42]. They observed that the the failure rate was 10.3% in the one-stage revision group and 6.1% in the two-stage group. In additional analyses, 24% (12/49) of patients were newly found to have concomitant knee injuries (e.g., chondral defects, meniscal lesions) at the time of the second-stage operative procedure.

Diermeier et al. [43] reported the results of 54 patients who underwent bone grafting due to recurrent, symptomatic ACL deficiency following ACLR. Only 44 patients underwent a staged revision ACLR after bone grafting and 10 patients refused to undergo a revision ACLR. At a mean period of 33.9 months, there was an improvement in the Lysholm score (77.2 ± 15.5 vs 72.9 ± 18.7), IKDC score (69.0 ± 13.4 vs 69.3 ± 13.4) and Tegner activity score (4.1 ± 1.5 vs 4.6 ± 1.2) for both groups. But no significant difference was observed between the two groups. Knee-laxity measurements were elevated in the without-revision group, but the difference was not significant. Postoperatively, no complications were reported and none of the included patients had a flexion or extension deficit. However, the small number of included patients, especially in the group of patients without revision ACLR, is limited.

## Conclusions

In active young patients, failed primary ACLR may require a revision ACLR. Two-stage revision ACLR should be considered in cases of tunnel lysis, infection, malalignment, meniscal deficiency, or chondral lesions. A two-stage procedure is technically more demanding than the primary or one-stage procedure and outcomes are potentially inferior, especially for active patients who make a high demand on their bodies. However, with precise indications, proper preoperative planning and operative-technique selection, two-stage revision ACLR can achieve favorable outcomes.

## Data Availability

Not applicable, this is a review article.

## Abbreviations

ACL: Anterior cruciate ligament
ACLR: Anterior cruciate ligament reconstruction
ALL: Anterolateral ligament
sCO2: Supercritical carbon dioxide
Si-CaP: Silicate-substituted calcium phosphate
